# Evaluation of survival extrapolation in immuno-oncology using multiple pre-planned data cuts: learnings to aid in model selection

**DOI:** 10.1186/s12874-020-00997-x

**Published:** 2020-05-06

**Authors:** Ash Bullement, Anna Willis, Amerah Amin, Michael Schlichting, Anthony James Hatswell, Murtuza Bharmal

**Affiliations:** 1 Delta Hat, Nottingham, UK; 2 BresMed, Sheffield, UK; 3 Merck Serono Ltd, Feltham, UK; 4 Merck KGaA, Darmstadt, Germany; 5 Department of Statistical Science, University College London, London, UK; 6 Oncology Brands & Life Cycle Management, Global Evidence & Value Development, EMD Serono, Inc, One Technology Place, Rockland, MA 02370 USA

**Keywords:** Survival, Extrapolation, Cancer, Immune-oncology, Immunotherapy

## Abstract

**Background:**

Due to limited duration of follow up in clinical trials of cancer treatments, estimates of lifetime survival benefits are typically derived using statistical extrapolation methods. To justify the method used, a range of approaches have been proposed including statistical goodness-of-fit tests and comparing estimates against a previous data cut (i.e. interim data collected). In this study, we extend these approaches by presenting a range of extrapolations fitted to four pre-planned data cuts from the JAVELIN Merkel 200 (JM200) trial. By comparing different estimates of survival and goodness-of-fit as JM200 data mature, we undertook an iterative process of fitting and re-fitting survival models to retrospectively identify early indications of likely long-term survival.

**Methods:**

Standard and spline-based parametric models were fitted to overall survival data from each JM200 data cut. Goodness-of-fit was determined using an assessment of the estimated hazard function, information theory-based methods and objective comparisons of estimation accuracy. Best-fitting extrapolations were compared to establish which one provided the most accurate estimation, and how statistical goodness-of-fit differed.

**Results:**

Spline-based models provided the closest fit to the final JM200 data cut, though all extrapolation methods based on the earliest data cut underestimated the ‘true’ long-term survival (difference in restricted mean survival time [RMST] at 36 months: − 1.1 to − 0.5 months). Goodness-of-fit scores illustrated that an increasingly flexible model was favored as data matured. Given an early data cut, a more flexible model better aligned with clinical expectations could be reasonably justified using a range of metrics, including RMST and goodness-of-fit scores (which were typically within a 2-point range of the statistically ‘best-fitting’ model).

**Conclusions:**

Survival estimates from the spline-based models are more aligned with clinical expectation and provided a better fit to the JM200 data, despite not exhibiting the definitively ‘best’ statistical goodness-of-fit. Longer-term data are required to further validate extrapolations, though this study illustrates the importance of clinical plausibility when selecting the most appropriate model. In addition, hazard-based plots and goodness-of-fit tests from multiple data cuts present useful approaches to identify when a more flexible model may be advantageous.

**Trial registration:**

JAVELIN Merkel 200 was registered with ClinicalTrials.gov as NCT02155647 on June 4, 2014.

## Background

Immuno-oncology treatments aim to exploit the body’s immune system to target and kill cancer cells [1]. Different ‘immunotherapy’ classes have been studied in a range of cancers, though perhaps one of the most notable advances in contemporary medicine has been the development of immune-checkpoint inhibitors [1–3]. Immune-checkpoint inhibitors were first licensed for use in melanoma, followed by a number of other cancers including non-small-cell lung cancer, renal cell carcinoma and urothelial carcinoma [4]. More recent approvals have included the use of immune-checkpoint inhibitors in rare cancers, such as microsatellite instability-high or mismatch repair deficient solid tumors [4].

Clinical trials facilitate the collection of data regarding the safety and efficacy of the intervention(s) under study however data collection is subject to a number of limitations. These include the number and characteristics of patients recruited, the generalizability of the study design to clinical practice, and the duration over which data are collected. The latter of these limitations (known as administrative censoring) plays a key role when establishing the clinical- and cost-effectiveness of interventions. There is a growing trend in accelerated or conditional approvals and breakthrough designations being granted by the Food and Drugs Administration (FDA) and the European Medicines Agency (EMA) meaning it is more often the case that interim analyses are used to inform regulatory submissions, which are subsequently updated as further data are collected [5, 6].

Long-term outcomes are typically uncertain at the time of both regulatory and reimbursement assessment. Survival extrapolation is often used to estimate longer-term outcomes in support of reimbursement applications, which typically consider outcomes over a patient’s lifetime, however parametric estimates of survival (permitting inspection of both short- and long-term survival) have also factored into regulatory decisions [7, 8]. Establishing a robust estimate of OS for patients treated with immune-checkpoint inhibitors is of increased importance versus conventional systemic anticancer therapies, as a substantial proportion of the treatment benefit is anticipated to manifest in the longer term (i.e. beyond the duration follow-up typically available at the time of regulatory or reimbursement assessment).

Guidance for undertaking survival analysis of patient-level data is available from a number of sources; including the commonly-cited National Institute for Health and Care Excellence (NICE) Decision Support Unit (DSU) Technical Support Document (TSD) 14 [9–16]. TSD14 offers practical, transparent guidance for undertaking survival analysis regardless of therapeutic area and/or the mechanistic properties of interventions being assessed. Within TSD14, it is noted that the most popular types of survival extrapolation models submitted for review by NICE are parametric survival models (PSMs), which are commonly used internationally [9, 16–18]. PSMs assume the underlying survivor function may be represented by a statistical distribution; ranging in both complexity and flexibility, which may be compared using standard statistical tests. PSMs do not require any specific assumptions to be fitted, though the appropriateness of a specific PSM for a given data set may be determined through an interrogation of the patient-level data; hence PSMs are a popular choice of extrapolation method to inform submissions of evidence to regulatory and reimbursement agencies.

In this study, we present a range of PSMs to predict OS beyond the observed period in a case study clinical trial (JAVELIN Merkel 200 [JM200] of avelumab [Bavencio®] for patients with metastatic Merkel cell carcinoma [mMCC], NCT02155647) [19]. PSMs were fitted to four pre-planned published trial data cuts in order to establish how predictions changed over time, and assess the accuracy of initial projections versus data later made available. By re-fitting and testing the accuracy of model predictions, it is possible to retrospectively identify emergent evidence of likely long-term survival outcomes and, by extension, inform best modelling practice.

## Methods

### Motivating example

The motivating example used in this study was the single-arm (i.e. uncontrolled), Phase II JM200 trial of avelumab for the treatment of mMCC. Including the data submitted to the FDA, a total of four distinct pre-planned data cuts from Part A of the JM200 trial (conducted in treatment-experienced mMCC) have been published, providing information regarding the evolving pattern of OS as data from the trial mature. Within the context of this study, each data cut refers to the minimum follow-up data available for all patients that are still being followed up for OS within the study – e.g. a “12-month” data cut refers to the interim data collected up until all patients had been followed up until at least 12 months (though some patients may have been followed up for longer). The term “pre-planned interim analysis” has also been used to describe different data cuts in other studies.

Data from JM200 offer a unique opportunity to retrospectively assess the accuracy of survival projections over a number of data cuts, particularly when acknowledging that at the time data from JM200 were published, survival outcomes for patients with mMCC receiving standard care were poor (median OS of approximately 5.3 months for patients with distant mMCC following second-line chemotherapy in Europe) [20–22]. Consequently, little was known around the likely long-term outcomes associated with avelumab treatment in an mMCC population. The key features of these data cuts are summarized in Table 1.

**Table 1:** Data cuts from Part A of the JAVELIN Merkel 200 clinical trial

Label	Database lock	Minimum patient follow-up	Source(s)
12mo	September 3, 2016	12 months	Kaufman et al., (2018) [23]
18mo	March 24, 2017	18 months	D’Angelo et al., (2018) [24]
24mo	September 26, 2017	24 months	Nghiem et al., (2018) [25]
36mo	September 14, 2018	36 months	D’Angelo et al., (2020) [26]

### Assessment of data

Patient-level data from each data cut were assessed following guidance from TSD14, from which suitable PSMs were identified for fitting. TSD14 recommends the use of hazard-based plots to inform appropriate model selection. As data from the (single-arm) JM200 trial are only available for patients receiving avelumab, some aspects of TSD14 are irrelevant (e.g. testing for proportional hazards between multiple treatment groups).

All analyses were performed using the statistical software *R* [27]. The package ‘muhaz’ was used to produce smoothed hazard estimates to aid selection of appropriate PSMs. Smoothed hazard plots provide an illustration of how the estimated hazard of death changes over time, allowing for inference to be made around which PSMs would be expected to provide a good fit to the data, and thus yield plausible survival estimates. PSMs were rejected where the smoothed hazard plots demonstrated a clear violation of the model functional form – for example, were the smoothed hazard plot to demonstrate a monotonically-increasing pattern of hazards over time, the exponential model (which assumes a constant hazard rate) would be rejected.

Empirical hazard plots (e.g. number of events per month) have been considered in a previous study as an alternative representation of the estimated hazard function (where time is considered on a continuous scale), however these plots would have limited use to inform appropriate model selection within the context of the JM200 trial due to its small sample size (*n* = 88) [28]. This is because there will be several periods over which the hazard of death would be estimated as zero as no events may have occurred within a given timeframe. Smoothed hazard plots are not affected by this issue to the same extent, hence were preferred for this study.

### Fitted models

The focus of this study was on the use of PSMs that do not require implicit or explicit assumptions regarding the patient population, disease area, or therapeutic class of the intervention. As such, two different types of PSMs were fitted: [1] standard PSMs, and [2] Royston and Parmar spline-based PSMs [29]. The *R* package ‘flexsurv’ was used to fit both standard and spline-based PSMs [30]. Other modelling approaches (such as cure-based or mixture models) were not considered as these require the estimation and/or specification of mixing weights or cure probabilities. No specific parametric modelling approaches were pre-specified in the JM200 study protocol, and so while each of the modelling approaches may be considered post-hoc analyses, this is not unusual with the context of survival extrapolation.

The standard PSMs considered were the exponential, Weibull, Gompertz, lognormal, log-logistic, and generalized gamma, in line with guidance from TSD14. These PSMs are commonly used as the range of candidate PSMs in economic evaluations of cancer interventions. As discussed previously, the exponential PSM assumes a constant hazard rate over time, whereas both the Weibull and Gompertz PSMs assume a monotonically increasing or decreasing hazard rate over time (excluding the special case of the Weibull wherein the shape parameter = 1, in which case it is equivalent to the exponential PSM). The lognormal, log-logistic, and generalized gamma models do not assume a monotonic hazard rate over time, and as such are able to reflect turning points in the underlying hazard function. TSD14 provides a more detailed summary of each of the standard PSMs.

Spline-based PSMs use natural, cubic, piecewise polynomials to smooth between sections of a transformation of the baseline survivor function.^1^ The number of sections is based on a specified number of ‘knots’ (equivalent to cut-points), and the fit within each section is based on a selected functional form. A detailed explanation of spline-based PSMs is provided by Royston and Parmar (2002) [29]; though to summarize, Royston and Parmar suggest the use of these flexible parameterizations to better reflect the “behavior” of the hazard rate over time.

In our study, we fitted spline-based PSMs assuming functional forms that are extensions of the Weibull, log-logistic and log-normal standard PSMs – henceforth referred to as hazard, odds, or normal spline-based PSMs, respectively. The models were fitted with the same intention as per the standard PSMs – that is, to provide a parametric estimate of the survivor function over time. The spline-based PSMs were fitted with 1, 2, or 3 internal knots, considered to provide a sufficiently broad number of alternative models to choose between, avoiding the use of more than 3 knots (equivalent to more than 4 degrees of freedom) as the output may be unstable [29]. Knot locations were selected according to the percentiles of the log-uncensored survival times (as previous research has shown the determination of knot locations does not appear critical for good fit) [31, 32].

Based on the selection of PSMs deemed appropriate (following the assessment of the underlying hazard function), a comparison of PSMs was undertaken to determine those providing the ‘best fit’ to the trial data. The determination of best-fitting models is (to an extent) subjective, and so a range of methods were explored covering statistical goodness-of-fit and prediction accuracy independent of model complexity.

### Statistical goodness of fit

Four statistical goodness-of-fit scores were considered, described in turn within Table 2, as well as the unadjusted maximized log-likelihood. The maximized log-likelihood was considered as a simplistic representation of the model providing the best fit to the data, without any penalty considered with regards to the complexity of the model fitted. For comparison to other measures of statistical fit, the maximized log-likelihood was multiplied by − 2 (henceforth termed −2*LL*). It is noted that within TSD14 that the use of the −2*LL* statistic should only be considered when comparing nested models [9]. The presentation of the −2*LL* statistic is therefore provided primarily for context, such that the relative penalties for complexity of other statistical goodness-of-fit scores may be inferred.

**Table 2:** Measures of statistical goodness-of-fit

Acronym	Full name	Formula
−2*LL*	−2 × *maximised log* − *likelihood*	−2*LL* = 2 log(*L*)
*AIC*	*Akaike information criterion*	*AIC* = 2*k* − 2 log(*L*)
*AICc*	*Akaike information criterion* (*corrected*)	$AICc=2k-2\mathit{\log}(L)+\frac{2{k}^2+2k}{n-k-1}$
*HQC*	*Hannan* – *Quinn information criterion*	*HQC* = 2*klog*(*log*(*n*)) − 2 *log* (*L*)
*BIC*	*Bayesian information criterion*	*BIC* = log(*n*)*k* − 2 log(*L*)

**Key:***k* Number of model parameters; *L* Maximized likelihood function; *log* Natural logarithm; *n* Number of data points (sample size)

Akaike and Bayesian information criteria (*AIC* and *BIC*, respectively) were calculated, which are well documented in published literature, and described in detail within TSD14 [9]. Due to the relatively-small sample size of JM200, a corrected version of the *AIC* (*AICc*) was also considered – literature suggested the *AICc* may be relevant to consider when the ratio of the sample size and number of model parameters is < 40 (in our example, this would apply for PSMs with 3 or more parameters). Finally, the Hannan–Quinn information criterion (*HQC*) was calculated; which has been cited in a number of studies to date, yet received little attention within the context of PSMs [33, 34]. Within the context of this study (where *n* = 88), the *HQC* considers a penalty for model complexity between the *AIC* and *BIC* – the per-parameter penalty is approximately 3.0 for the *HQC*, versus 2.0 and 4.5 for the *AIC* and *BIC*, respectively.

### Prediction accuracy

Previous studies which have attempted to assess the prediction accuracy of PSMs within the context of cancer immunotherapy have considered a range of techniques. Ouwens et al. considered a combination of statistical goodness-of-fit and area-under-the-curve estimates [28]. Bullement et al. presented a range of point estimates at specific time points relative to the maturity of data from each study [35].

For completeness, two summary statistics were considered: [1] the Kaplan-Meier (KM) versus modelled point-estimate of survival, and [2] the restricted-mean survival time (RMST) derived via area-under-the-curve (for the KM versus predicted survival). Point-estimates provide a simple representation of modelled survival accuracy at specific timepoints. The RMST has previously been proposed as an alternative to the conventional hazard ratio used in the design of randomized controlled trials with a time-to-event outcome, and is broadly aligned with the expected outcome of economic modelling (i.e. if survival curves are used to inform a cost-effectiveness analysis, the estimation of life-years is based on an area-under-the-curve calculation) [36].

Summary statistics were considered at key timepoints relating to the maturity of each data cut. The minimum and maximum follow-up time for each patient was considered for each data cut, as well as the mid-point between these times. The resultant timepoints corresponded to (approximately) 6-monthly intervals from 12 to 48 months. Estimated survival at 36 months is of particular importance as this is the latest point in time for which the KM estimate of survival may be considered fixed due to all living patients having been followed up for at least 36 months.

In addition to these objective measures of prediction accuracy, a visual assessment of PSM fit was also considered. For simplicity, the most notable estimates are provided within this article, and an exhaustive presentation of each model is provided as supplementary material.

## Results

### Assessment of data

**Fig. 1:**
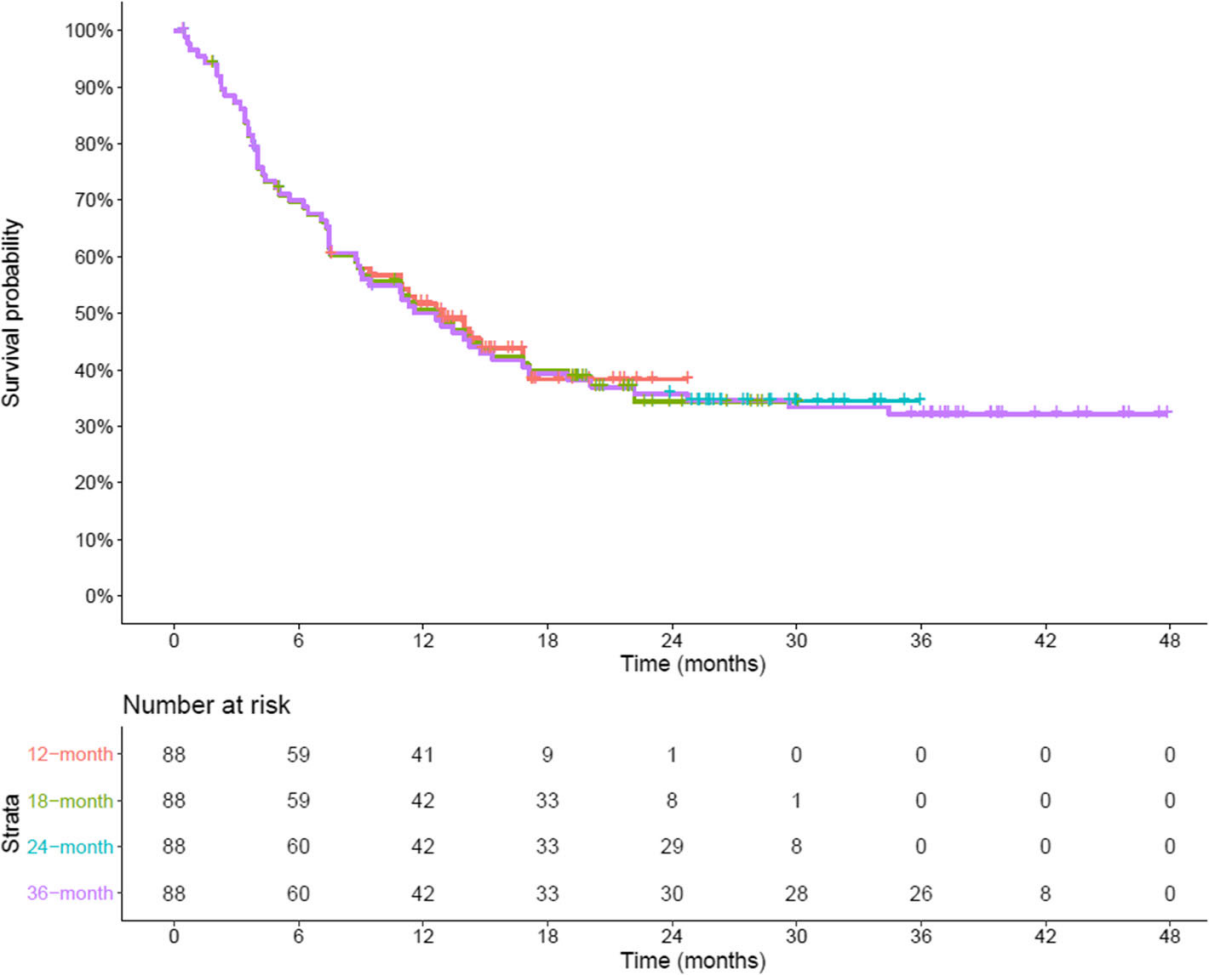
Overall survival data from Part A of the JAVELIN Merkel 200 clinical trial. **Key:** mFU, minimum follow-up; mo, month(s)

The available data from each of the four data cuts of the JM200 trial are presented as KM curves Fig. 1. Over time a plateau in the OS curve has emerged, indicating that the specification of a PSM incapable of reflecting time-varying hazards is unlikely to yield a good fit to the available data, and consequently would not be expected to provide a plausible extrapolation. Of particular note is the number of patients at risk for each data cut at specific points in time – while estimates of 2.5-year (30-month) OS for the two latest data cuts were within 2% of each other (34.5 and 33.4%, for the 24- and 36-month data cuts, respectively); the number of patients at risk at this time in the later data cut is noticeably larger (*n* = 8 versus *n* = 28).

**Fig. 2:**
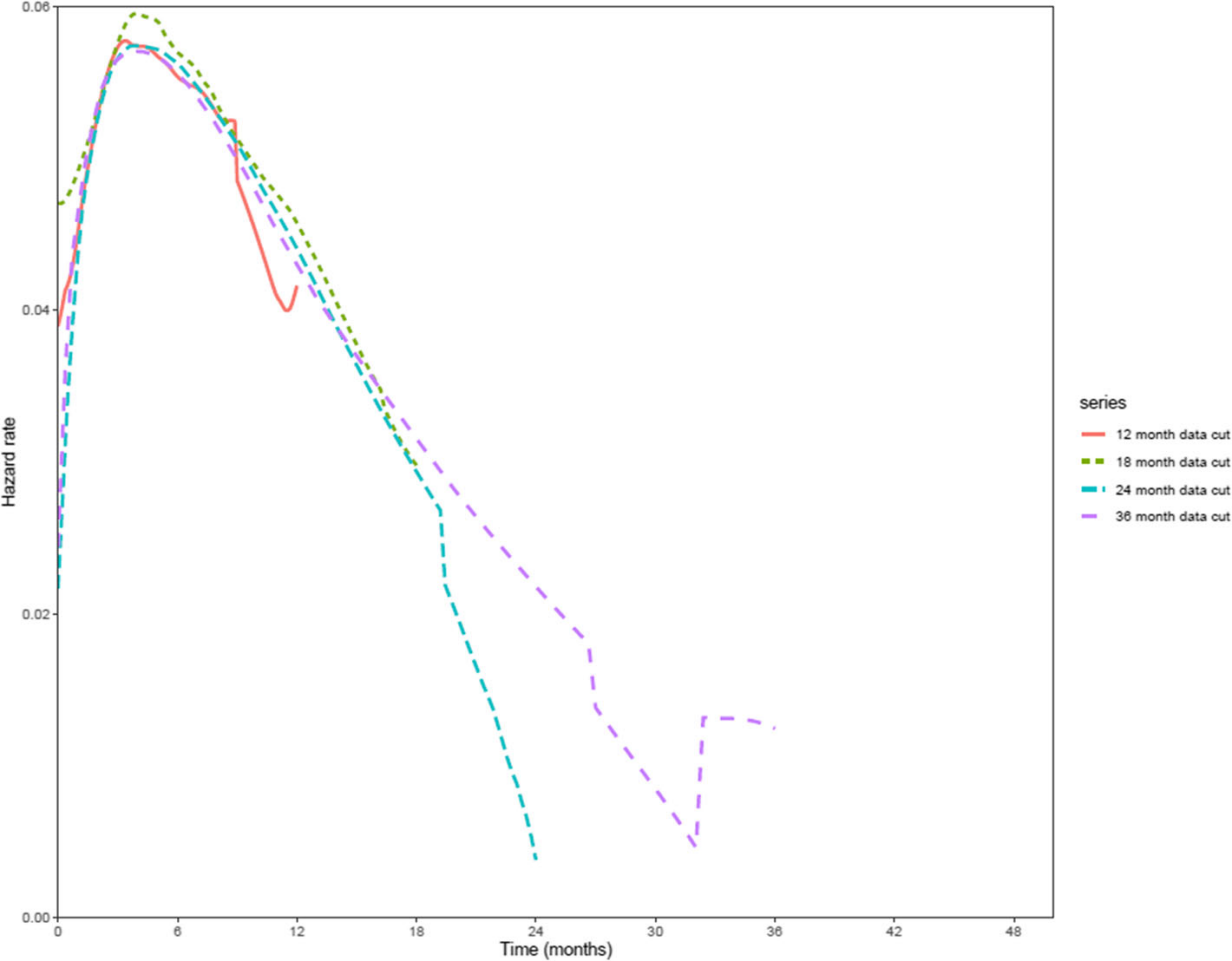
Smoothed hazard plots from Part A of the JAVELIN Merkel 200 clinical trial. **Note:** Owing to the sample size of JAVELIN Merkel 200 Part A (*n* = 88 patients), the *max.time* argument required by the *muhaz* function was set to the minimum follow-up time for each data cut. Consequently, the smoothed hazard estimate for each data cut is presented within this figure for a limited time period

The smoothed hazard plots produced for each data cut are provided in Fig. 2. As expected, the plots for each data cut exhibit a non-constant hazard rate over time. Further to this, the hazard function appears to be non-monotonic (i.e. the hazard appears to increase and then decrease). Based on this assessment, the lognormal, log-logistic and generalized gamma PSMs would be expected to provide a reasonable fit to the data, as would the spline-based PSMs. However, the exponential, Weibull, and Gompertz PSMs are unlikely to provide a plausible OS extrapolation, due to the relative inflexibility of these models; and were therefore not considered further.

### Statistical goodness of fit

The statistical goodness-of-fit scores for each of the PSMs are presented in Table 3. Unsurprisingly, the non-penalized −2*LL* score demonstrated a clear preference for the more flexible 3-knot spline-based PSMs across all four data cuts, given that these models have the most parameters and thus may be considered to have the greatest freedom to best fit to data. The standard lognormal PSM was shown to have the lowest goodness-of-fit scores of all PSMs fitted to the 12- and 18-month data cuts. Other PSMs shown to provide a good fit were the log-logistic, 1-knot hazard, and 1-knot odds models.

**Table 3:** Statistical goodness-of-fit scores of fitted models

Statistic	Model	12mo mFU	18mo mFU	24mo mFU	36mo mFU
**-2LogL**	Log-logistic	375.3	429.4	453.5	483.0
	Log-normal	373.7	427.5	451.3	480.5
	Gen Gamma	373.3	426.5	448.7	475.0
	1-knot Hazard	373.3	426.5	448.0	471.9
	1-knot Odds	373.3	426.3	447.8	472.5
	1-knot Normal	373.4	426.6	448.8	474.2
	2-knot Hazard	373.4	426.5	447.4	470.8
	2-knot Odds	373.3	426.3	447.3	471.3
	2-knot Normal	373.2	426.2 (2)	447.2	471.1
	3-knot Hazard	372.7 (3)	426.4	446.6 (2)	469.6 (1)
	3-knot Odds	372.5 (2)	426.3 (3)	446.7 (3)	469.9 (3)
	3-knot Normal	372.2 (1)	426.1 (1)	446.6 (1)	469.8 (2)
**AIC**	Log-logistic	379.3	433.4	457.5	487.0
	Log-normal	377.7 (1)	431.5 (1)	455.3	484.5
	Gen Gamma	379.3	432.5	454.7 (3)	481.0
	1-knot Hazard	379.3 (3)	432.5 (3)	454.0 (2)	477.9 (1)
	1-knot Odds	379.3 (2)	432.3 (2)	453.8 (1)	478.5 (2)
	1-knot Normal	379.4	432.6	454.8	480.2
	2-knot Hazard	381.4	434.5	455.4	478.8 (3)
	2-knot Odds	381.3	434.3	455.3	479.3
	2-knot Normal	381.2	434.2	455.2	479.1
	3-knot Hazard	382.7	436.4	456.6	479.6
	3-knot Odds	382.5	436.3	456.7	479.9
	3-knot Normal	382.2	436.1	456.6	479.8
**AICc**	Log-logistic	379.4 (2)	433.5	457.6	487.2
	Log-normal	377.8 (1)	431.7 (1)	455.5	484.7
	Gen Gamma	379.6	432.8	455.0 (3)	481.3
	1-knot Hazard	379.6	432.8 (3)	454.3 (2)	478.2 (1)
	1-knot Odds	379.5 (3)	432.6 (2)	454.1 (1)	478.8 (2)
	1-knot Normal	379.6	432.9	455.0	480.5
	2-knot Hazard	381.9	434.9	455.9	479.3 (3)
	2-knot Odds	381.8	434.8	455.8	479.7
	2-knot Normal	381.7	434.7	455.7	479.6
	3-knot Hazard	383.4	437.2	457.3	480.3
	3-knot Odds	383.2	437.0	457.4	480.6
	3-knot Normal	382.9	436.8	457.3	480.6
**HQC**	Log-logistic	381.3 (2)	435.4 (3)	459.5	489.0
	Log-normal	379.7 (1)	433.5 (1)	457.3 (3)	486.5
	Gen Gamma	382.3	435.5	457.7	484.0
	1-knot Hazard	382.3	435.5	457.0 (2)	480.9 (1)
	1-knot Odds	382.3 (3)	435.3 (2)	456.8 (1)	481.5 (2)
	1-knot Normal	382.4	435.6	457.8	483.2
	2-knot Hazard	385.4	438.5	459.4	482.8 (3)
	2-knot Odds	385.3	438.3	459.3	483.2
	2-knot Normal	385.2	438.2	459.2	483.1
	3-knot Hazard	387.7	441.4	461.6	484.6
	3-knot Odds	387.4	441.3	461.7	484.9
	3-knot Normal	387.2	441.1	461.6	484.8
**BIC**	Log-logistic	384.2 (2)	438.4 (2)	462.4	492.0
	Log-normal	382.7 (1)	436.5 (1)	460.3 (1)	489.5
	Gen Gamma	386.8	440.0	462.1	488.4
	1-knot Hazard	386.7	440.0	461.4 (3)	485.3 (1)
	1-knot Odds	386.7 (3)	439.7 (3)	461.2 (2)	485.9 (2)
	1-knot Normal	386.8	440.0	462.2	487.7 (3)
	2-knot Hazard	391.3	444.4	465.3	488.7
	2-knot Odds	391.2	444.2	465.2	489.2
	2-knot Normal	391.1	444.1	465.1	489.0
	3-knot Hazard	395.1	448.8	469.0	492.0
	3-knot Odds	394.8	448.7	469.1	492.3
	3-knot Normal	394.6	448.5	469.0	492.2

**Key:***AIC* Akaike information criterion; *AICc* Akaike information criterion (corrected); *BIC* Bayesian information criterion; Hannan–Quinn information criterion; *L* Maximized likelihood function; *log* Natural logarithm; *mFU* Minimum follow up; *mo* Month(s)

**Note:** For each of the scores presented above, a lower value indicates a better statistical goodness-of-fit. The “best” fitting model (i.e. the model with the lowest score) is denoted with “(1)” after the score, and is shaded in dark grey. Models with ranks 2 and 3 are formatted similarly

Each of the four goodness-of-fit criteria (*AIC*, *BIC*, *AICc*, and *HQC*, which trade-off model fit and complexity) were generally in agreement, though the *AIC* (which has the lowest penalty for model complexity) was shown to exhibit an ‘earlier’ preference (with regards to comparing scores across the four data cuts) for a spline-based PSM (the second-best AIC from the 12- and 18-month data cuts was for the 1-knot odds PSM). In the latest (36-month) data cut, the preferred PSM measured by all four criteria was the 1-knot hazard spline. Notably, even some of the 2- and 3-knot spline-based PSMs yielded a better statistical goodness-of-fit than the lognormal PSM fitted to the 36-month data cut (which was considered the best-fitting PSM in the earlier data cuts).

### Prediction accuracy

**Fig. 3:**
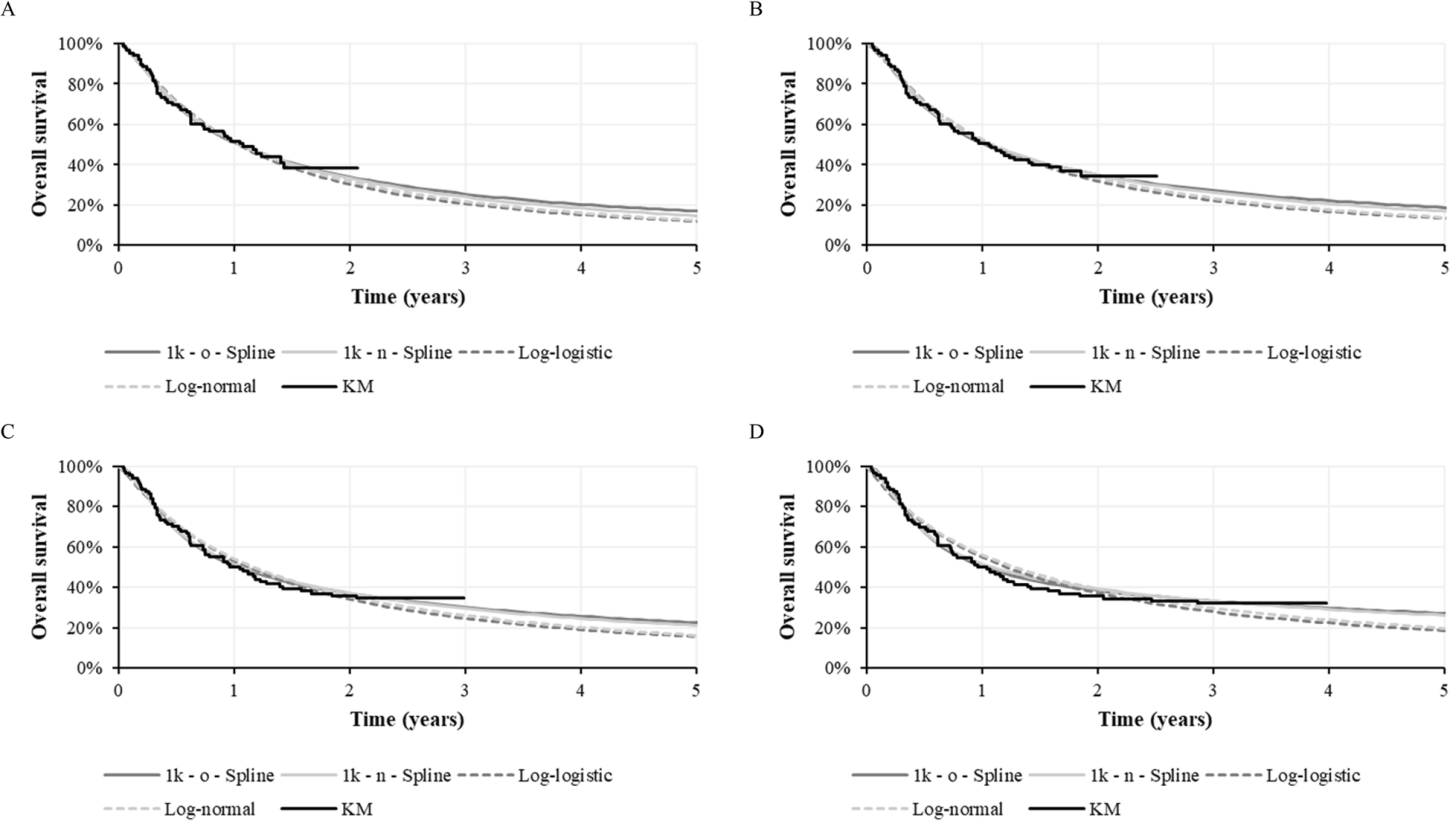
Fitted models from Part A of the JAVELIN Merkel 200 clinical trial**. Notes: A**, 12-month data cut; **B**, 18-month data cut; **C**, 24-month data cut; **D**, 36-month data cut. **Key:** k, knot(s); KM, Kaplan-Meier; n, normal; o, odds.

To compare the prediction accuracy of models of differing complexity over each data cut, the lognormal, log-logistic, 1-knot odds, and 1-knot normal PSMs were selected for consideration. These models were selected owing to their statistical goodness-of-fit scores, as well as the fact that the odds and normal spline-based PSM are extensions to the log-logistic and log-normal standard PSMs, respectively. These models are provided for each data cut in Fig. 3 for a timeframe of 5 years.

Visual inspection of the PSMs fitted to each data cut demonstrates increasingly greater estimates of longer-term OS, which is unsurprising as the maturing data from JM200 show an increasingly clearer plateau in the KM curve. In addition, a comparison of the best fitting standard and spline-based PSMs for each data cut show spline-based PSMs consistently provide estimates closer to the ‘true’ OS, although both under-estimate OS as demonstrated in the 36-month data cut.

An excerpt of the prediction accuracy results based on the summary statistics are provided in Table 4 (complete results are provided in the supplementary appendix). When comparing PSM fits from the three earlier data cuts to the KM curve for the latest data-cut, it may be inferred that none of the models (standard or spline-based) provided an estimate of 3-year survival greater than or equal to the ‘true’ value of approximately 32.1%. The closest fit was achieved using the 24-month data cut by the 3-knot hazard spline (31.7%). The model fitted to the 12-month data cut which yielded the closest estimates to 36-month survival was the 1-knot odds spline-based PSM (25.5%). These findings conflict with the output of the statistical goodness-of-fit statistics, which suggest the spline-based PSMs may over-fit to the data.

**Table 4:** Prediction accuracy key findings

Data cut	Model	Criteria for model selection	Prediction accuracy (months)			
			PE		RMST	
			Fitted (earlier) data cut	Latest (36-mo) data cut	Fitted (earlier) data cut	Latest (36-mo) data cut
12-mo	KM estimates		**51.8**	**32.1**	**8.7**	**17.5**
12-mo	Log-normal	Best AIC, BIC	−0.3	−10.2	−0.2	−0.9
12-mo	Log-logistic	Lowest RMST, PE	−0.7	−11.5	−0.2	−1.2
12-mo	1-knot Odds	Highest RMST, PE	−0.8	−6.6	−0.3	−0.5
18-mo	KM estimates		**39.9**	**32.1**	**11.3**	**17.5**
18-mo	Log-normal	Best AIC, BIC	+ 1.1	−8.7	+ 0.0	−0.5
18-mo	Log-logistic	Lowest RMST, PE	+ 0.0	−9.8	− 0.0	− 0.8
18-mo	1-knot Odds	Highest RMST	+ 1.2	−5.1	− 0.2	− 0.4
18-mo	3-knot Odds	Highest PE	+ 1.1	−4.8	−0.2	− 0.4
24-mo	KM estimates		**35.8**	**32.1**	**13.5**	**17.5**
24-mo	1-knot Odds	Best AIC	+ 1.2	−2.0	+ 0.0	+ 0.0
24-mo	Log-normal	Best BIC	−0.1	−6.0	+ 0.4	+ 0.1
24-mo	Log-logistic	Lowest RMST, PE	−1.6	−7.4	+ 0.2	−0.3
24-mo	Gen Gamma	Highest RMST	+ 1.2	−2.7	+ 0.2	+ 0.1
24-mo	3-knot Hazard	Highest PE	+ 0.9	−0.4	−0.1	− 0.1
36-mo	KM estimates		**32.1**	**32.1**	**17.5**	**17.5**
36-mo	1-knot Hazard	Best AIC, BIC, highest PE	+ 1.4	+ 1.4	+ 0.2	+ 0.2
36-mo	3-knot Hazard	Lowest RMST	+ 0.9	+ 0.9	−0.1	−0.1
36-mo	Log-normal	Highest RMST	−2.3	− 2.3	+ 0.9	+ 0.9
36-mo	Log-logistic	Lowest PE	−4.1	−4.1	+ 0.4	+ 0.4

**Key:***AIC* Akaike’s information criterion; *BIC* Bayesian information criterion; *Gen* Generalized; *mo* Month(s); *PE* Point estimate; *RMST* Restricted mean survival time

**Note:** Negative values indicate that the model underestimates survival, whereas positive values indicate that the model overestimates survival. ‘Fitted’ refers to the data cut from which the models were fitted (i.e. the data cut stated within the left-hand column), and so a comparison is made between a model fitted to a given data cut and the Kaplan-Meier curve for this same data cut. ‘Latest’ refers to the 36-month data cut, and so a comparison is made between a model fitted to the specified data cut (which may be earlier) and the Kaplan-Meier curve for the 36-month data cut. Models were included in this table if one or more of the following criteria were met: (1) the model provided the ‘best’ AIC or BIC score, (2) the model provided either the ‘highest’ or ‘lowest’ estimate of RMST at 36 months, or (3) the model provided either the ‘highest’ or ‘lowest’ PE of survival at 36 months. Where RMST estimates were tied (to the nearest 0.1 month), the model with the lowest AIC or BIC was included here. Full prediction accuracy results are provided within the supplementary material

Based on the earlier two data cuts, the modelled and KM-estimated RMST values are broadly comparable taken at the maximum follow-up time for each data cut (of the ‘best fitting’ models, the largest under-estimate was 0.3 months). However, when comparing the same models with the latest (36-month) KM curve, the spline-based models provided a closer fit (spline-based models under-estimated 36-month RMST [17.5 months] by 0.4–0.5 months, versus 0.5–1.2 months for the standard parametric models). For the 24-month data cut, the standard parametric models over-estimated RMST by 0.2–0.4 months, whereas for the 36-month data cut, the standard parametric models over-estimated RMST by as much as 0.9 months.

Based upon the original 12-month data cut, each of the PSMs predicted 12-month survival within 1% of the ‘true’ value. Estimated 24-month survival ranged from 30.2% (log-logistic) to 34.0% (1-knot odds spline), whereas the ‘true’ value (revealed in later data cuts) was 35.8% (with a preliminary estimate from the 12-month data cut of 38.4%). The log-logistic and 1-knot odds spline models based on the 12-month data cut both provided similar statistical goodness-of-fit scores (AIC and BIC scores within 2 points of each other). Therefore, based on the clinical expectation of a survival plateau, similar statistical goodness-of-fit scores, and the prediction accuracy results based on the summary statistics presented, it may be reasonable to select the 1-knot odds spline model. In later data cuts, this model was shown to provide an under-estimate of survival, yet minimized the under-estimation of survival versus all other options fitted based on the 12-month data cut (including the log-logistic).

## Discussion

This study presents an application and subsequent validation of parametric survival modelling of multiple pre-planned data cuts, using data from a case study of avelumab for mMCC. Four data cuts were utilized to demonstrate how initial projections were affected when refitted with more complete data. While standard PSMs had the best statistical goodness-of-fit score in earlier data cuts (determined from all four formal goodness-of-fit criteria presented in our study); for later data cuts, more flexible spline-based PSMs provided the ‘best’ scores, as well as a more accurate estimation of the pattern of survival over time. A broader view of statistical goodness-of-fit scores therefore appears critical in determining the best-fitting model.

To date, a number of studies have attempted to establish the prediction accuracy of extrapolation methods used for immune-checkpoint inhibitors. Gibson et al., (2017) validated the extrapolation of spline-based PSMs to predict progression-free survival for advanced melanoma patients treated with ipilimumab, nivolumab, or the combination of the two treatments enrolled within the CheckMate 067 trial using external data from patients treated with ipilimumab monotherapy [37]. Bullement et al., (2019) also performed a validation of survival extrapolation techniques in advanced melanoma, using re-created data from two data cuts of the pivotal ipilimumab CA184–024 trial [38]. Ouwens et al., (2019) explored a broad range of extrapolation methods using data for patients with non-small-cell lung cancer from the ATLANTIC trial of durvalumab [28].

Within the context of our case study, Lanitis et al., (2019) presented a range of alternative extrapolation approaches, including landmark analyses based on response and progression status [39]. Due to data availability at the time of analysis, each of these studies were conducted using only two data cuts (i.e. one data cut for estimation, and a second for validation). As the majority of studies have been conducted in melanoma, it is unclear whether or not the findings are generalizable to other cancer types, particularly as the possibility of long-term survival for melanoma patients was established prior to the introduction of immune-checkpoint inhibitors – historical estimates of 10-year survival for Stage IV melanoma patients ranged from 7 to 20% (dependent on metastatic site) [40, 41].

This study makes use of four formal, pre-planned data cuts from the same registrational trial. The availability of several data cuts from the same study allows for a more in-depth assessment of appropriate PSM fits versus previous studies wherein only one additional data cut is usually available, without the need to generalize across different studies (e.g. by comparing to registry and/or historical control data). By comparing the PSMs and corresponding statistical goodness-of-fit scores across each data cut, an emergent picture may be ascertained regarding which PSM could be reasonably selected.

When fitting the PSMs, published guidance NICE DSU TSD14 was followed, and a systematic approach to appropriate model selection was adopted. In addition to standard models, spline-based PSMs were also fitted to provide a broad range of survival estimates. While to date these models have not been used extensively, previous studies have highlighted the potential role of flexible PSMs (including spline-based PSMs) within the context of complex hazard functions (which may be due to a combination of the disease area, mechanistic properties of the intervention, or clinical trial study design) [28, 32, 38, 39]. In addition to exploring a range of extrapolation methods, this study illustrates the value of assessing the available trial data via hazard plots in order to inform appropriate model selection. The use of hazard plots and/or other diagnostic plots is advocated in available guidance (including TSD14), though these plots are rarely used to their full potential [9].

Used in combination with hazard-based plots, the potential importance of understanding and interpreting statistical goodness-of-fit scores was highlighted by our study. Where goodness-of-fit scores disagree and/or different PSMs exhibit scores within close proximity to one another, it may be useful to explore further why this is the case (and thus infer if there is a clear reason to favor one model or score over the other). Burnham and Anderson (2002) highlight a ‘rule of thumb’ concerning the *AIC*, which states that if the difference between the best and an alternative PSM is ≤2 points, there is *“substantial empirical support”* for the alternative, poorer-fitting PSM (and so this model should not necessarily be rejected based on the AIC alone) [34]. Hilbe (2011) offers a slightly different rule of thumb, noting that if the difference is ≤2.5 points there is *“no difference”* in the models; and if the difference is ≤6.0 points, the alternative PSM should only be rejected if the sample size *n* > 256 [42].

In our motivating example, the sample size was *n* = 88, and the lognormal PSM was preferred for the two earlier data cuts. However, there was emergent evidence of the next best-fitting model (1-knot odds spline-based PSM) providing a reasonable fit, and a potentially more accurate estimate of survival. The difference in *AIC* for the models was approximately 1.6, illustrating the importance of looking beyond the best-fitting model, should evidence be available to suggest doing so (in our example, this may be based on the more accurate estimate of the RMST up until 24 months). While potentially challenging to interpret while data are maturing, we believe the interpretation of statistical goodness-of-fit scores is currently an under-used tool that may help aid selection of appropriate models outside of simply choosing the model with the lowest score. However, statistical goodness-of-fit scores only reflect the goodness-of-fit within the observed period, and so should not be considered as a comprehensive representation of overall model fit.

Further to the notion of statistical goodness-of-fit scores potentially being an underused tool in model selection, the choice of statistical fit score to inform model selection is seldom discussed. Literature notes that both the *BIC* and *HQC* are not true estimators of Kullback–Leibler (KL) divergence (which is essentially a measure of how one probability distribution [in our case, the distribution of survival times estimated via a PSM] is different from another), and are instead focused upon the selection of the ‘true’ model which exists and is within the set of fitted PSMs being considered [34]. Conversely, the *AIC* and *AICc* are focused upon the identification of the model that minimizes the KL divergence of the model and the ‘true’ underlying function being estimated (for which the ‘true’ model does not necessarily exist) [34].

While a relatively subtle difference in model interpretation, given the context of our motivating example (that is, increasingly maturing data cuts which are expected to gradually reveal a more accurate estimate of the underlying survivor function), it would seem more appropriate to consider goodness-of-fit scores that do not require the assumption of a ‘true’ model existing and being present within a set of models to choose between. More specifically, simple parametric models fitted to preliminary data cuts might not consider specific characteristics of the ‘true’ survival pattern that is impacted by the maturity of data, delayed treatment effects, the potential for long-term survivors, and other relevant real-world aspects. We recommend the choice of goodness-of-fit score to inform model selection should therefore be determined within the context of the underlying decision problem to ascertain which score(s) may be most appropriate under specific circumstances. Alternatively, consideration of a broad range of scores may aid model selection (including the lesser-used *HQC*, which like the *AIC* exhibited a preference for the 1-knot odds spline based on the 24-month data cut, yet the *BIC* did not), given that it is often the case that only *AIC* and *BIC* are considered in submission to NICE (based on TSD14 guidance) [9].

The importance of appropriate survival extrapolation is particularly highlighted within the context of HTA, as noted within NICE TSD 14: *“different methods have varying functional forms and are likely to result in different survival estimates, with the differences potentially large – particularly when a substantial amount of extrapolation is required”* [9]. A model under-estimating RMST by approximately 1 month would translate to an under-estimate in life-years gained of 0.08, equivalent to approximately 0.06 (undiscounted) QALYs (assuming a utility value of 0.71 per the published cost-utility analysis of avelumab in mMCC) [43]. Though a seemingly small decrease in QALYs gained, were this decrement applied to the published base-case cost-utility results, the incremental cost-effectiveness ratio (ICER) would increase by approximately £1000. The ICER would increase further were the extrapolated portion of the curve also considered to under-estimate survival markedly (as may be expected given the under-estimate of the RMST).

NICE TSD 14 comments further on the difficulty in justifying the plausibility of the extrapolated portion of the survival model chosen, noting that this is likely to greatly influential on the estimated mean survival. It is recommended that model choice is based on the use of external data sources, biological plausibility, or clinical expert opinion. In the context of our motivating example, no external data sources were available to inform model selection, and so clinical expert opinion and biological plausibility have increased importance when selecting between alternative models.

Furthermore, we focused solely on the use of biological plausibility and clinical expert opinion as a means of selecting from a suite of models that had already been fitted (as opposed to factoring this information within the model fitting itself) – models that make use of external information within the model fitting process may also be important to consider, though were beyond the scope of our research question. An example of such approaches includes the relative survival framework, for which Dickman and Coviello (2015) present several worked examples within the context of population-based cancer registries [44]. Using this approach cause-specific survival is estimated relative to a comparable group from the general population. While this approach may yield improved estimates of survival, the data requirements are increased (through the need to specify a comparable group) and longer-term cause-specific hazards may still be difficult to estimate.

There are a number of alternative extrapolation methods that were not considered within our study. Lanitis et al. considered alternative models based on separating the population and/or survival outcomes based on intermediate outcome assessment (i.e. progression or response) [39]. Othus et al., (2017) demonstrated the potential role of mixture-cure modelling, wherein a proportion of patients are expected to be ‘statistically-cured’, and are subject to a hazard of death per the age- and sex-adjusted general population [45]. In addition to mixture-cure models, Ouwens et al., (2019) note the potential role of other mixture models (which do not assume a ‘cured’ fraction) and Lanitis et al. presented landmark analyses that may improve prediction accuracy) [28, 39]. While these techniques may yield reasonable extrapolations, the focus of our study was on the use of purely parametric approaches such that any differences in fit and/or long-term estimation may be considered as a factor of the model selected (and not any other decisions, such as the existence of distinct patient groups, or clinically-relevant timepoints). Furthermore, statistical goodness-of-fit scores should not be compared for models which utilize different sources of external information (such as background mortality rates), hence the omission of these models within our study allows for a valid comparison of statistical goodness-of-fit scores.

Data from the JM200 trial allow for a comparison of PSM projection accuracy up to at least 36 months (per the minimum follow-up time for patients in the latest data cut), beyond which extrapolations remain. Obtaining repeated data cuts from clinical trials is subject to practical limitations, and so while further data collection is necessary to truly determine the most plausible extrapolation technique within this case study, we may never truly be able to validate the entire projection of OS. Plans for the publication of repeated data cuts should be specified within the design of clinical trials with survival-based endpoints where possible, in order to further understand how patterns of survival may change over time.

## Conclusions

The findings of the study show that while more flexible models (such as spline-based PSMs) may offer sub-optimal statistical goodness-of-fit scores in early data cuts (due to the penalties applied for model complexity), they may be able to more accurately reflect emergent complex hazard functions, provide estimate more closely aligned with biological plausibility/ clinical expert opinion, and consequently yield more credible longer-term survival estimates. As such, a thorough exploration of PSMs outside of the standard six PSMs is encouraged where complex hazard functions are expected, as well as a detailed exploration of statistical goodness-of-fit scores and their interpretation.

While data from the JM200 trial are specific to mMCC patients treated with avelumab, the implications of the analysis performed using our motivating example may be useful more broadly when choosing between alternative survival extrapolation methods – that is, further inspection of statistical goodness-of-fit scores specifically may aid understanding of the likely pattern of survival as interim data mature. We urge the preferred selection of survival extrapolation to be based on a multitude of factors including statistical goodness-of-fit, visual fit, biological plausibility, hazard plots and other relevant diagnostic plots, and encourage the use of multiple data cuts (both earlier and later) and clinical expert opinion to select and validate extrapolations where possible.

## Abbreviations

-2LL: -2 multiplied by the maximized log-likelihood
AIC: Akaike’s information criterion
AICc: Akaike’s information criterion (corrected)
BIC: Bayesian information criterion
DSU: Decision support unit
EMA: European medicines agency
FDA: Food and drug administration
HQC: Hannan-Quinn information criterion
JM200: JAVELIN merkel 200
KM: Kaplan-Meier
mMCC: Metastatic Merkel cell carcinoma
NICE: National institute for health and care excellence
OS: Overall survival
PSM: Partitioned-survival model
RMST: Restricted mean survival time
TSD: Technical support document

## Footnotes

1 Natural (or ‘restricted’) splines limit the estimation of the model within the boundary knots (as extending beyond these boundaries is generally understood to lead to a poorer fit). Cubic splines consider a polynomial of order 3 – the smallest order which allows an inflexion in the transformation of the survivor function.
